# The Effects of Pomegranate Supplementation on Markers of Exercise-Induced Muscle Damage: A Systematic Review and Meta-Analysis

**DOI:** 10.1016/j.cdnut.2025.104560

**Published:** 2025-01-28

**Authors:** Saba Belyani, Fatemeh Kazeminasab, Mahnaz Niazi, Reza Bagheri, Mahsa Mahabadi Hesari, Sara K Rosenkranz, Donny M Camera, Fred Dutheil

**Affiliations:** 1 Department of Human Sciences, Human Nutrition Program, The Ohio State University, Columbus, OH, United States; 2 Department of Physical Education and Sport Sciences, Faculty of Humanities, University of Kashan, Kashan, Iran; 3 Department of Nutrition, Varastegan Institute for Medical Sciences, Mashhad, Iran; 4 Department of Exercise Physiology, University of Isfahan, Isfahan, Iran; 5 Department of Exercise Physiology, Faculty of Sport Science, Alzahra University, Tehran, Iran; 6 Department of Kinesiology and Nutrition Sciences, University of Nevada Las Vegas, Las Vegas, NV, United States; 7 Department of Health and Biostatistics, Swinburne University, Melbourne, Australia; 8 Université Clermont Auvergne, CNRS, LaPSCo, Physiological and Psychosocial Stress, CHU Clermont-Ferrand, University Hospital of Clermont-Ferrand, Preventive and Occupational Medicine, Witty Fit, Clermont-Ferrand, France

**Keywords:** fruit, nutrition, exercise, recovery, nutritional supplements

## Abstract

**Background:**

Pomegranate supplementation has been shown to reduce oxidative stress and inflammation, with some evidence suggesting it may accelerate recovery from exercise-induced muscle damage (EIMD), including metabolic, mechanical, and neuromuscular recovery.

**Objectives:**

This systematic review and meta-analysis aimed to investigate the effects of pomegranate supplementation on markers of EIMD.

**Methods:**

A systematic search of Scopus, PubMed, and Web of Science up to January 2024 identified studies evaluating pomegranate supplementation and exercise recovery. Studies involving athletes and nonathletes aged 18–55 were included. Weighted mean differences (WMDs) were calculated for EIMD markers. Study quality was assessed using a modified physiotherapy evidence database scale. This review was registered with the International Prospective Register of Systematic Reviews (ID: CRD42024536905).

**Results:**

Ten studies were included in the meta-analysis. Pomegranate supplementation did not significantly affect markers of metabolic recovery, including myoglobin (WMD: –1.344 ng/mL; 95% confidence interval (CI): –4.11, 1.42 ng/mL, *P* = 0.342) and creatine kinase (WMD: –11.990 U/L; 95% CI: –28.64, 4.66 U/L, *P* = 0.158), or neuromuscular recovery, as indicated by lactate concentrations (WMD: –0.093 mmol/L; 95% CI: –0.39, 0.21 mmol/L, *P* = 0.546). Muscle soreness also remained unchanged (WMD: 0.999; 95% CI: –0.18, 2.17, *P* = 0.097). However, a significant reduction in lactate dehydrogenase amounts (WMD: –21.152 U/L; 95% CI: –39.29, –3.01 U/L, *P* = 0.022) immediately postexercise suggests a short-term protective effect against mechanical muscle damage.

**Conclusions:**

Pomegranate supplementation does not appear to enhance overall recovery markers for EIMD but may offer short-term benefits for mechanical muscle damage. Standardizing supplementation regimens, dosages, and exercise protocols is crucial to better understand the potential benefits of pomegranate supplementation in EIMD recovery.

## Introduction

Effective recovery strategies are essential for optimizing performance, maintaining training consistency, and reducing risk of injury [1]. Intense, unaccustomed exercise often leads to oxidative stress, inflammation, and exercise-induced muscle damage (EIMD), which can hinder progress and adherence to training programs[2,3]. Vigorous exercise typically results in muscle weakness and soreness, peaking 24–48 h postexercise and sometimes lasting several days [2]. Eccentric exercise, in particular, is associated with significant EIMD, leading to delayed-onset muscle soreness, strength loss, and prolonged recovery periods [4,5]. These changes are largely attributed to oxidative stress and inflammation caused by muscle tissue damage [6]. Addressing oxidative stress and inflammation has been shown to accelerate recovery and improve skeletal muscle function [7].

Nutritional strategies with antioxidant and anti-inflammatory properties are increasingly explored as effective recovery aids [8]. Polyphenols, a class of naturally occurring antioxidants found in plants, have attracted attention for their potential benefits in sports performance and recovery [9]. These compounds, found in foods such as red wine, green tea, and pomegranate, have been associated with enhanced endurance, muscle power, maximal oxygen consumption (VO_2max_), and recovery, with performance improvements of ≤1.90% reported [[10], [11], [12]]. Among polyphenol-rich foods, pomegranate stands out for its potent antioxidant activity and unique bioactive compounds, such as punicalagin, ellagic acid, and anthocyanins [13,14]. Notably, its antioxidant activity is ∼3 times greater than that of red wine or green tea, making it particularly effective in reducing oxidative stress and inflammation and enhancing nitric oxide bioavailability to improve blood flow [15,16]. Beyond its sport-specific benefits, pomegranate is considered a “superfood” due to its ability to reduce risk factors for chronic diseases, including high blood pressure, hyperglycemia, and inflammation [17]. The proposed mechanisms for these benefits include scavenging reactive oxygen species, reducing lipid peroxidation, modulating proinflammatory cytokines like IL-6 and TNF-α, and promoting vasodilation through enhanced nitric oxide bioavailability [[18], [19], [20]]. In the context of exercise, pomegranate supplementation has been linked to improved strength recovery and reduced muscle soreness after eccentric exercise [21]. Although systematic reviews have highlighted its potential benefits, no meta-analysis has specifically evaluated its effects on markers of EIMD [22,23].

Given these promising properties, this systematic review and meta-analysis aim to systematically evaluate the effects of pomegranate supplementation on markers of EIMD, including metabolic, mechanical, and neuromuscular recovery, as well as systemic inflammation. Metabolic markers, such as myoglobin and creatine kinase (CK), reflect muscle fiber damage and energy metabolism restoration [24]. Mechanical recovery, assessed using lactate dehydrogenase (LDH), pertains to structural muscle integrity, whereas neuromuscular recovery, evaluated through lactate concentrations, reflects fatigue and metabolic restoration [24,25]. Additionally, C-reactive protein (CRP) is included as a systemic marker of inflammation [24,26]. By categorizing and evaluating these biomarkers, this study provides a comprehensive understanding of pomegranate’s role in supporting recovery and its potential as a natural aid for performance enhancement.

## Methods

### Study registration

The present systematic review and meta-analysis were carried out according to the guidelines set by the PRISMA [27]and the Cochrane Handbook of Systematic Reviews of Interventions [28]. Additionally, this study was registered in advance in the PROSPERO (ID: CRD42024536905).

### Search strategy

A comprehensive electronic database search was conducted in Scopus, PubMed, and Web of Science up to 1 January 2024 by 2 independent researchers. The keywords “Pomegranates” or “Pomegranate” or “Punicagranatum” or “Punicagranatums” or “punica granatum” and “Exercise” or “training” or “Exercise training” or “Physical Activity” or “eccentric exercise” or “strength training” or “resistance training” or “endurance training” or “strenuous exercise” or “exercise performance” or “muscle performance” or “sports performance” or “muscle recovery” or “sports fatigue” or “athletes” and “Muscle soreness” or “muscle damage” “delayed-onset muscle soreness” or “range of motion” or “perceived soreness” or “rate of perceived exertion” or “maximum voluntary contraction” or “creatine kinase” or “myoglobin” or “troponin” or “lactate dehydrogenase” or “blood urea nitrogen” or “serum creatinine” or “alanine aminotransferease” or “serum glutamic-pyruvic transaminase” or “aspartate aminotransferase” or “serum glutamic-oxaloacetic transaminase” or “recovery” or “injury∗” or “muscle∗” or “perform∗” or “recover∗” or “muscle enzyme activity” were used to conduct the searches. Articles were exported from databases to EndNote. Duplicate articles were removed and screened with EndNote. To ensure comprehensive capture of relevant records, the reference lists of all included studies were hand-searched for any additional sources that may have been missed in the initial electronic search. Articles were searched based on title and abstract. The search was limited to articles written in English and studies involving athletes and nonathletes. There was no limit on publication dates.

In cases where the 2 independent researchers disagreed on the inclusion of a study, a third researcher was consulted to resolve the disagreement. This approach ensured a balanced and objective decision-making process, enhancing the reliability of the study selection.

### Study selection and inclusion criteria

Studies that involved adult humans (athletes and nonathletes) consuming a pomegranate supplement either “after exercise” or “before and after exercise” were included if they met the following criteria: *1*) the study design was a randomized controlled trial (RCT), nonrandomized, and crossover intervention; *2*) outcomes were muscle soreness and/or proxy markers of muscle damage such as myoglobin, CK, LDH, lactate, and CRP; and *3*) authors measured ≥1 outcome at baseline and immediately after, and/or 2, and/or 24, and/or 48, and/or 72 h after an acute exercise. The following exclusion criteria were applied: *1*) studies that co-administered drugs (pharmaceutical compounds) and/or other supplements; *2*) studies without a control group; and *3*) participants with any metabolic diseases, cardiovascular, or musculoskeletal disorders.

### Quality assessment and sensitivity analyses

The quality assessment of potential bias was conducted using the physiotherapy evidence database (PEDro) scale [29]. We used the PEDro scale to assess the risk of bias as an indicator of quality. This is an accepted tool for assessing quality in clinical trials. We excluded 2 of the 11 items (due to no blinding of the participants and researchers) since participants and intervention providers were not blinded to the assigned supplementation and exercise training conditions during interventions. Therefore, our study employed a scale comprising 9 items: *1*) defined criteria for eligibility, *2*) randomly assigned participant allocation, *3*) concealed allocation, *4*) similarity of groups at baseline, *5*) blinding of all assessors, *6*) evaluated outcomes in 85% of participants, *7*) intention-to-treat (ITT) analysis, *8*) reporting of statistical comparisons between groups, and *9*) point measures and measures of variability. Sensitivity analyses were also conducted for all outcomes by the “remove 1” technique. Such a procedure assesses whether individual studies had a disproportionate effect on the results of the meta-analyses. The quality assessment of the studies was performed by the first (SB) and third authors (MN). In case of disagreement, the second author (FK) was consulted.

### Statistical analysis

Statistical analyses were completed using comprehensive meta-analysis version 2.0 software. These analyses involved calculating weighted mean difference (WMD), along with 95% confidence intervals (CIs), to assess outcomes. Analyses were performed using random-effects models. Effect sizes were computed to assess and compare the effects of pomegranate supplementation on muscle soreness and/or markers of EIMD. To assess heterogeneity, the I^2^ statistic was calculated, with a significance level set at *P* < 0.05. Per the guidelines provided by Cochrane, the interpretation of I^2^ statistics was as follows: 25% indicated low heterogeneity, 50% indicated moderate heterogeneity, and 75% indicated high heterogeneity [30]. The findings considered the possibility of heterogeneity of both clinical and/or methodological factors that could have influenced the outcomes [31]. Publication bias was identified by analyzing funnel plots. In cases of publication bias, Egger’s tests were employed as a supplementary confirmatory measure. If the *P* value of the Egger’s test was <0.1, an observable publication bias was indicated [32]. Subgroup analyses were performed according to the timing of the outcome measurements after an acute exercise (immediately, 2 h, 24 h, 48 h, or 72 h).

## Results

### Included studies

**FIGURE 1:**
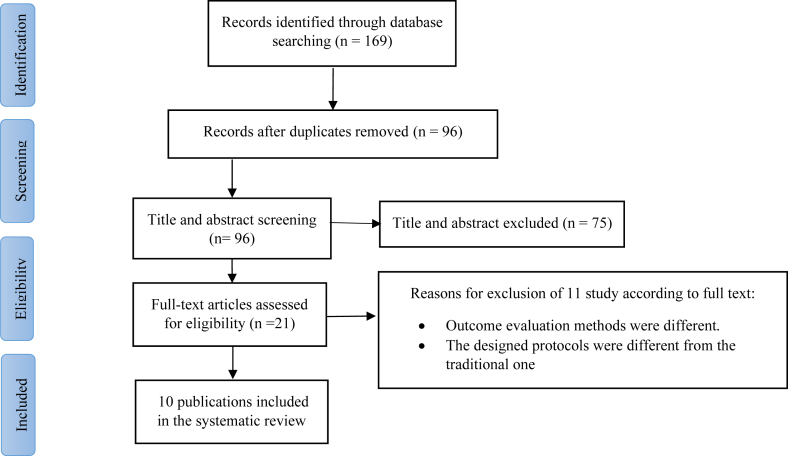
Flow diagram of systematic literature search.

The initial search strategy yielded a total of 40 records from PubMed, 69 records from Scopus, and 50 records from Web of Science. After excluding duplicate records and assessing the titles and abstracts, 21 studies were determined to meet eligibility requirements and required a comprehensive assessment of the complete texts. After conducting a detailed assessment of the full text, 11 studies were excluded for the following reasons: they did not measure muscle soreness, myoglobin, CK, LDH, or lactate. In this systematic review and meta-analysis, a total of 10 studies were assessed, which comprised 10 intervention groups involving pomegranate supplementation and acute exercise. A flowchart diagram referring to the systematic literature search is displayed in Figure 1.

Two studies were RCTs [33,34], 3 were nonrandomized studies [[35], [36], [37]], and 5 were crossover study designs [2,20,21,38,39]. Supplementation regimens of pomegranate ranged from 20 mL [33] to 1500 mL [35] from 1 to 8 wk in duration. In the crossover studies, the washout period between each of the conditions ranged from 7 d to 14 d (Supplemental Table 1).

Muscle soreness was reported in 5 studies. All articles calculated muscle soreness using a visual analog scale. The proxy markers of EIMD were reported in the studies as follows: muscle soreness was reported in 5 studies, myoglobin in 3 studies, CK in 7 studies, LDH in 3 studies, lactate in 4, and CRP in 4 studies.

Based on the observations for each outcome, all eligible studies included different timing for outcome assessments. Most of the included studies reported multiple assessment time points (i.e., immediately postexercise (0 h), 2 h, 24 h, 48 h, and 72 h after exercise).

Muscle soreness was assessed pre-exercise, 2 h, 24 h, 48 h, and 72 h after exercise, and myoglobin was assessed pre-exercise, 0 h, 24 h, 48 h, and 72 h after exercise, and CK and CRP were measured 0 h, 2 h, 24 h, 48 h, and 72 h after exercise, and LDH and lactate concentrations were measured pre-exercise and 0 h.

### Participant characteristics

A combined 198 participants (149 athletes and 49 nonathletes) were included in the overall analysis, with sample sizes ranging from 8 [20] to 36 [34] adults. The mean age and BMI (in kg/m^2^) varied across studies. The mean ages ranged from 17 y [20] to 34.9 y [38], whereas the BMI ranged from 21.3 [33] to 25.6 ± 4.0 [34]. A total of 10 studies were included in the meta-analysis. Among them, 9 studies only included male participants [2,21,33,34,[36], [37], [38], [39], [40]], and 1 study included both male and female participants [20]. Eight studies included athletes [2,20,21,[33], [34], [35], [36],^38^], whereas 2 studies focused on healthy participants [37,39]. For more detailed participant characteristics, refer to Table 1 [2,20,21,[33], [34], [35], [36], [37], [38], [39]].

**TABLE 1:** Participant characteristics of included studies.

Source, year	Sample size (sex)	Type of study	Groups	Outcomes	Health and training status	Age (years) mean ± SD	BMI (kg/m^2^) mean ± SD
Torregrosa-Garcia et al., 2019 [38]	26 M	Crossover	Intervention (*n* = 13) Control (*n* = 13)	CK CRP Lactate	Amateur endurance-trained male athletes	Intervention: 34.9 ± 10.0 Control: 34.9 ± 10.0	Intervention: 24.5 ± 3.0 Control: 24.5 ± 3.0
Trombold et al., 2009 [21]	16 M	Crossover	Intervention (*n* = 8) Control (*n* = 8)	Muscle soreness Myoglobin CK CRP	Healthy, nonsmoking, and recreationally active males	Intervention: 24.2 ± 1.4 Control: 24.2 ± 1.4	NM
Ammar et al., 2016 [35]	9 M	NRS	Intervention (*n* = 9) Control (*n* = 9)	CK CRP LDH Muscle soreness	Elite weightlifters	Intervention: 21 ± 0.5 Control: 21 ± 0.5	NM
Pranskuniene et al., 2020 [33]	18 M	RCT	Intervention (*n* = 9) Control (*n* = 9)	Lactate	Healthy and recreationally active	Intervention: 22–28 Control: 22–28	Intervention: 21.3 (20.7–27.3) Control: 21.9 (19.8–25.3)
May Crum et al., 2017 [20]	8 M and F	Crossover	Intervention (*n* = 4) Control (*n* = 4)	Lactate	Highly-trained cyclists	Intervention: 17–18 Control: 17–18	NM
L. Lamb et al., 2019 [34]	36 M	RCT	Intervention (*n* = 12) Control (*n* = 12)	Muscle soreness CK	Healthy, nonresistance-trained males	Intervention: 24.0 (IQR 22.0–33.0) Control: 24.0 (IQR 22.0–33.0)	Intervention: 25.6 ± 4.0 Control: 25.6 ± 4.0
Martinez-Sancheza et al., 2017 [39]	19 M	Crossover	Intervention (*n* = 19) Control (*n* = 19)	Muscle soreness Myoglobin LDH CK	Healthy male subject	Intervention: 23.9 ± 3.7 Control: 23.9 ± 3.7	NM
Urbaniak et al., 2018 [36]	19 M	NRS	Intervention (*n* = 10) Control (*n* = 9)	CK Myoglobin Lactate	Polish rowing team	Intervention: 20.8 ± 0.86 Control: 20.9 ± 0.95	NM
Trombold et al., 2011 [2]	17 M	Crossover	Intervention (*n* = 17) Control (*n* = 17)	Muscle soreness	Resistance trained males	Intervention: 21.9 ± 2.4 Control: 21.9 ± 2.4	NM
Bayat-Chadegani et al., 2015 [37]	30 M	NRS	Intervention (*n* = 15) Control (*n* = 15)	CK CRP LDH	Healthy young male	Intervention: 22.40 ± 1.41 Control: 22.30 ± 1.56	NM

This table summarizes key details of the included studies, including sample size, participant demographics (e.g., sex, age, BMI), study design (e.g., RCT, NRS), intervention and control groups, health and training status, and assessed outcomes.

Abbreviations: BMI, body mass index; CK, creatine kinase; CRP, C-reactive protein; IQR, interquartile range; LDH, lactate dehydrogenase; NM, not mentioned; SD, standard deviation.

### Meta-analysis

#### Changes in muscle soreness after exercise

Based on 19 intervention arms, pomegranate supplementation did not significantly change muscle soreness pre- to postexercise (WMD: 0.99; 95% CI: –0.18, 2.17, *P* = 0.090), compared with a control during the follow-up periods. Among the included studies, there was significant heterogeneity in changes in muscle soreness following exercise (I^2^ = 93.94%, *P* = 0.001). Examination of funnel plots and Egger’s test results (*P* = 0.940) did not show publication bias. Additionally, sensitivity analysis conducted using the “remove 1” technique demonstrated that there were no changes in the effect size, significance of the findings, or the direction of the results.

**FIGURE 2:**
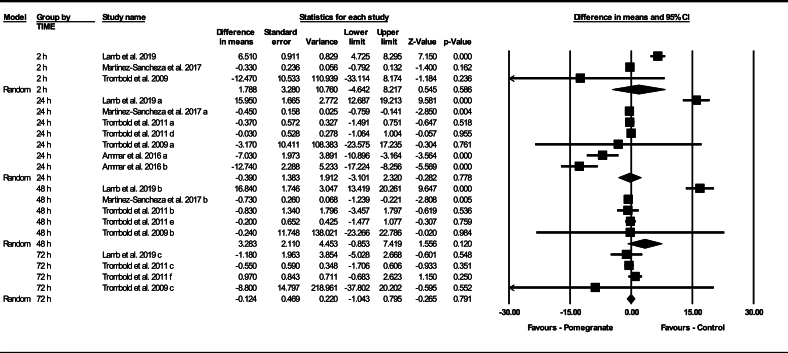
Forest plot of the impact of pomegranate supplementation compared with placebo on muscle soreness for 2 h, 24 h, 48 h, and 72 h after exercise. Data are reported as WMD (95% confidence limits). CI, confidence interval; WMD, weighted mean differences.

Subgroup analyses by the timing of the muscle soreness measurements revealed no significant change in muscle soreness pre- to postexercise for 2 h after (WMD: 1.788; 95% CI: –4.642, –8.217, *P* = 0.586, 3 interventions), 24 h after (WMD: –0.390; 95% CI: –3.101, 2.320, *P* = 0.778, 7 interventions), 48 h after (WMD: 3.283; 95% CI: –0.853, 7.419, *P* = 0.120, 5 interventions), or 72 h after exercise (WMD: –0.124; 95% CI: –1.043, 0.795, *P* = 0.791, 4 interventions), compared with a control group (Figure 2).

#### Changes in myoglobin after exercise

Based on 7 intervention arms, pomegranate supplementation did not decrease myoglobin pre- to postexercise (WMD: –1.34 ng/mL; 95% CI: –4.11, 1.42, *P* = 0.340 ng/mL), compared with a control during the follow-up periods. Among the included studies, there was significant heterogeneity (I^2^ = 77.04%, *P* = 0.001). Examination of funnel plots and Egger’s test results (*P* = 0.370) did not show publication bias. Additionally, sensitivity analysis conducted using the “remove 1” technique demonstrated that there were no changes in the effect size, significance of the findings, or the direction of the results.

**FIGURE 3:**
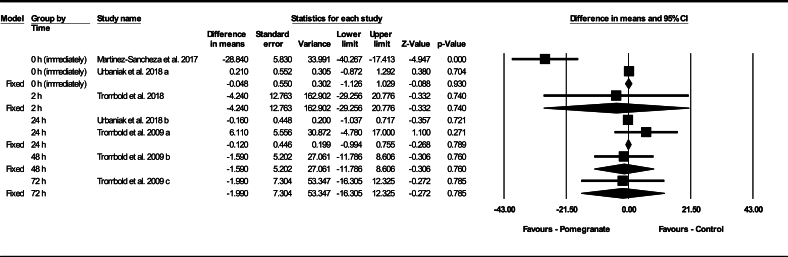
Forest plot of the impact of pomegranate supplementation compared with control on myoglobin for immediately, 2 h, 24 h, 48 h, and 72 h after exercise. Data are reported as WMD (95% confidence limits). CI, confidence interval; WMD, weighted mean differences.

Subgroup analyses by timing of the myoglobin measurements revealed no significant change in myoglobin pre- to postexercise at 0 h (WMD: –13.735 ng/mL; 95% CI: –42.181, 14.711 ng/mL, *P* = 0.344, 2 interventions), 2 h after (WMD: –4.240 ng/mL; 95% CI: –29.256, 20.776 ng/mL, *P* = 0.740, 1 intervention), 24 h after (WMD: 0.529 ng/mL; 95% CI: –3.315, 4.373 ng/mL, *P* = 0.787, 2 interventions), 48 h after (WMD: –1.590 ng/mL; 95% CI: –11.786, 8.606 ng/mL, *P* = 0.760, 1 intervention), or 72 h after (WMD: –1.990 ng/mL; 95% CI: –16.305, 12.325 ng/mL, *P* = 0.785, 1 intervention), compared with a control group (Figure 3).

#### Changes in CK after exercise

Based on 17 intervention arms, pomegranate supplementation did not decrease CK pre- to postexercise at 0 h (WMD: –11.99 U/L: 95% CI: –28.64, 4.66 U/L, *P* = 0.15), compared with control. Among the included studies, there was no significant heterogeneity (I^2^ = 38.55%, *P* = 0.05). Examination of funnel plots and Egger’s test results (*P* = 0.810) did not show publication bias. Additionally, sensitivity analysis conducted using the “remove 1” technique demonstrated that there were no changes in the effect size, significance of the findings, or the direction of the results.

**FIGURE 4:**
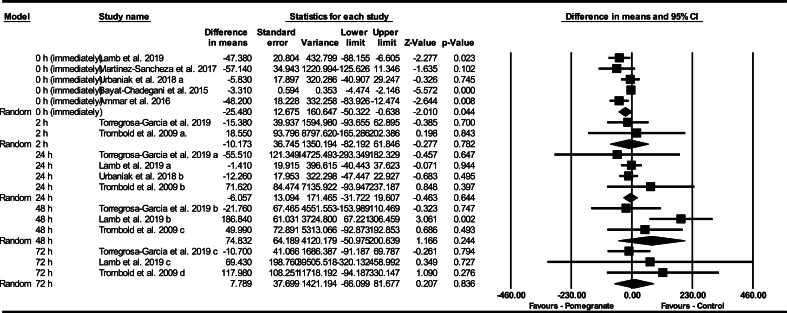
Forest plot of the impact of pomegranate supplementation compared with control on creatine kinase immediately, 2 h, 24 h, 48 h, and 72 h after exercise. Data are reported as WMD (95% confidence limits). CI, confidence interval; WMD, weighted mean differences.

Subgroup analyses by the timing of the CK measurements revealed a significant decrease in CK pre- to postexercise at 0 h (WMD: –25.48 U/L; 95% CI: –50.32, –0.63 U/L, *P* = 0.040, 5 interventions), but not at 2 h after (WMD: –10.17 U/L; 95% CI: –82.19, 61.84 U/L, *P* = 0.780, 2 interventions), 24 h after (WMD: –6.05 U/L; 95% CI: –31.72, 19.60 U/L, *P* = 0.640, 4 interventions), 48 h after (WMD: 74.83 U/L; 95% CI: –50.97, 200.63 U/L, *P* = 0.240, 3 interventions), or 72 h after exercise (WMD: 7.78 U/L; 95% CI: –66.09, 81.67 U/L, *P* = 0.830, 3 interventions), compared with a control group (Figure 4).

#### Changes in LDH after exercise

**FIGURE 5:**
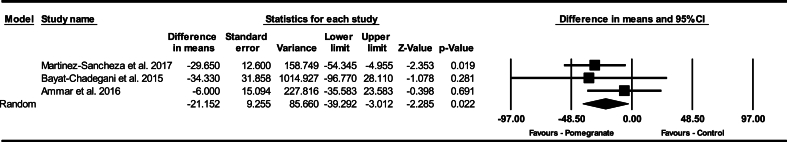
Forest plot of the impact of pomegranate supplementation compared with control on lactate dehydrogenase immediately after exercise. Data are reported as WMD (95% confidence limits). CI, confidence interval; WMD, weighted mean differences.

Based on 3 intervention arms, pomegranate supplementation decreased LDH pre- to postexercise at 0 h (WMD: –21.15 U/L; 95% CI: –39.29, –3.01 U/L, *P* = 0.020), when compared with a control (Figure 5). Among the included studies, there was no significant heterogeneity (I^2^ = 0.00%, *P* = 0.440). Examination of funnel plots and Egger’s test results (*P* = 0.920) did not show publication bias. Additionally, sensitivity analysis conducted using the “remove 1” technique demonstrated that there were no changes in the effect size, significance of the findings, or the direction of the results.

#### Changes in lactate after exercise

**FIGURE 6:**
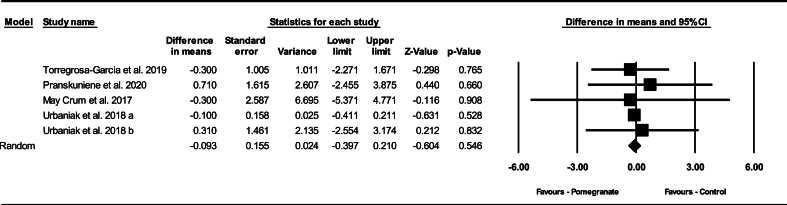
Forest plot of the impact of pomegranate supplementation compared with control on lactate immediately after exercise. Data are reported as WMD (95% confidence limits). CI, confidence interval; WMD, weighted mean differences.

Based on 5 intervention arms, pomegranate supplementation did not decrease lactate pre- to postexercise at 0 h (WMD: –0.09 mmol/L; 95% CI: –0.39, 0.21 mmol/L, *P* = 0.540), compared with a control (Figure 6). Among the included studies, there was no significant heterogeneity (I^2^ = 0.00%, *P* = 0.980). Examination of funnel plots and Egger’s test results (*P* = 0.480) did not show publication bias. Additionally, sensitivity analysis conducted using the “remove 1” technique demonstrated that there were no changes in the effect size, significance of the findings, or the direction of the results.

#### Changes in CRP after exercise

Based on 10 intervention arms, pomegranate supplementation did not change CRP pre- to postexercise immediately after exercise (WMD: –0.01 mg/L; 95% CI: –0.21, 0.19 mg/L), *P* = 0.900), compared with control. Among the included studies, there was no significant heterogeneity (I^2^ = 0.00%, *P* = 0.870). Examination of funnel plots and Egger’s test results (*P* = 0.930) did not show publication bias. Additionally, sensitivity analysis conducted using the “remove 1” technique demonstrated that there were no changes in the effect size, significance of the findings, or the direction of the results.

**FIGURE 7:**
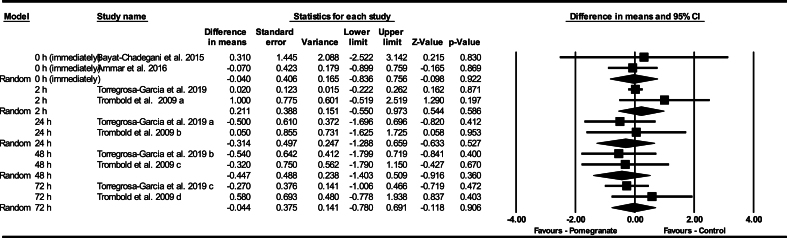
Forest plot of the impact of pomegranate supplementation compared with control on CRP immediately, 2 h, 24 h, 48 h, and 72 h after exercise. Data are reported as WMD (95% confidence limits). CI, confidence interval; CRP, C-reactive protein; WMD, weighted mean differences.

Subgroup analyses by measurement time revealed no significant change in CRP pre- to postexercise at 0 h (WMD: –0.04 mg/L; 95% CI: –0.83, 0.75 mg/L, *P* = 0.920, 2 interventions), 2 h after (WMD: 0.21 mg/L; 95% CI: –0.55, 0.97 mg/L, *P* = 0.580, 2 interventions), 24 h after (WMD: –0.31 mg/L; 95% CI: –1.28, 0.65 mg/L, *P* = 0.520, 2 interventions), 48 h after (WMD: –0.44 mg/L; 95% CI: –1.40, 0.50 mg/L, *P* = 0.360, 2 interventions), or 72 h after exercise (WMD: –0.04 mg/L; 95% CI: –0.78, 0.69 mg/L, *P* = 0.900, 2 interventions), compared with a control group (Figure 7).

### Quality assessment

The PEDro scale was used to assess the methodological quality by scoring the potential sources of bias for each individual study, with scores varying between 4 and 7 out of a possible maximum of 9 points. One study had a score of 7, 7 studies had scores of 6, and 2 studies had scores of 5. Most of the lower PEDro scores were due to 3 items (randomly assigned participants, concealed allocation, and ITT analysis). The details of the quality assessment are shown in Supplemental Table 2.

## Discussion

This systematic review and meta-analysis is the first to investigate the effects of pomegranate supplementation on muscle soreness and markers of EIMD. Although promising findings have suggested that pomegranate supplementation may have protective effects against EIMD, the overall results of this study indicate that pomegranate supplementation does not significantly influence most markers of EIMD, including muscle soreness, myoglobin, CK, lactate, and CRP. These findings are consistent with some RCTs that reported no significant effects of pomegranate supplementation on markers of EIMD [8,21,41]. Despite these results, there is evidence that athletes might benefit from pomegranate supplementation for muscle recovery due to other mechanisms not captured in the analyzed biomarkers.

Among the biomarkers assessed, LDH amounts were significantly reduced immediately after exercise, indicating pomegranate’s potential muscle-protective effects. LDH is a well-established marker of cellular damage, and its reduction suggests that pomegranate supplementation may help preserve muscle cell integrity by mitigating oxidative stress and inflammation. This finding aligns with previous studies reporting lower LDH amounts postexercise in pomegranate-supplemented groups [35,37,39,42]. However, given the limited number of intervention arms (*n* = 3) [35,37,39], these results should be interpreted cautiously and warrant replication in future studies with larger sample sizes. The reduction in LDH amounts observed in this study likely reflects pomegranate’s antioxidant, anti-inflammatory, and muscle-protective effects, which help maintain cellular integrity and minimize the leakage of LDH [43]. These findings underscore how pomegranate compounds can support tissue health and reduce markers of cellular damage [19]. Similarly, reductions in CK concentrations were observed immediately postexercise, reflecting acute protection against muscle damage. CK, another marker of EIMD, indicates structural muscle damage and repair processes. However, these effects were transient, with no significant differences observed at later time points. This finding underscores the importance of timing and suggests that pomegranate supplementation may provide short-term muscle-protective benefits. Future research should explore sustained supplementation strategies to prolong these effects and optimize recovery.

Myoglobin, an essential protein for oxygen delivery and storage in muscle cells, is another key marker of muscle damage. Although polyphenols may reduce myoglobin oxidation [44], previous studies and this meta-analysis found no significant changes in myoglobin concentrations with pomegranate supplementation. For instance, in rowers, myoglobin concentrations remained unchanged following supplementation [36]. Similarly, no changes were observed in non-resistance-trained males after eccentric exercise [43]. The lack of significant effects of pomegranate on myoglobin concentrations may be attributed to insufficient concentrations of bioactive compounds in the muscle tissue or low-induced oxidative damage that does not require substantial repair. Additional factors, such as supplementation timing, exercise protocol intensity, and participant characteristics (e.g., training status), may have contributed to these findings. Future studies should adjust dosages, timing, and study designs to clarify the relationship between pomegranate supplementation and myoglobin dynamics.

CRP, a systemic marker of inflammation, also did not exhibit significant changes in this study. Although pomegranate’s polyphenols, such as ellagic acid, are known for their anti-inflammatory properties, they may not be sufficient to influence systemic inflammation following EIMD. Factors such as supplementation dosage and duration, timing relative to exercise, and baseline inflammation levels likely play critical roles in determining efficacy [35]. The absence of significant changes in CRP could also suggest that pomegranate supplementation may be more effective in populations with elevated baseline inflammation rather than in healthy individuals engaging in acute exercise. Future studies should explore the use of higher doses, the inclusion of participants with elevated CRP concentrations, and alternative forms of pomegranate (e.g., fermented extracts or concentrated powders) to better understand its effects on systemic inflammation. Additionally, combining pomegranate with other anti-inflammatory interventions may amplify its benefits and warrant further exploration.

Lactate concentrations were similarly unaffected by pomegranate supplementation, corroborating findings from a previous systematic review that showed minimal effects on lactate clearance [23]. Lactate, a byproduct of anaerobic metabolism, is often used as an indicator of neuromuscular recovery, as its clearance reflects restored metabolic function [45]. Although lactate is not directly responsible for delayed-onset muscle soreness, its clearance provides insight into recovery processes [46,47]. The lack of significant effects on lactate concentrations suggests that pomegranate’s benefits are not primarily mediated through anaerobic metabolic recovery but may involve other pathways, such as oxidative stress reduction. Training adaptations, rather than supplementation alone, likely play a more substantial role in enhancing lactate clearance [40,48]. Future studies should investigate how supplementation impacts lactate dynamics in populations with varying exercise experience and fitness levels.

The lack of consistent effects of pomegranate supplementation on muscle recovery was unexpected, given its high content of ellagitannins and anthocyanins, which possess potent antioxidant and anti-inflammatory properties [8,40]. These compounds can scavenge reactive oxygen species generated during exercise, reducing lipid peroxidation and preserving cell membrane integrity. Furthermore, pomegranate’s modulation of proinflammatory cytokines, such as IL-6 and TNF-α, did not appear to influence systemic inflammation in this context [49]. Several factors may explain these inconsistencies, including heterogeneity in exercise protocols, participant characteristics, and supplementation regimens. For instance, differences in exercise type, intensity, and duration likely influenced the degree of EIMD and the observed effects of supplementation. Additionally, participant factors such as age, sex, and training status may have contributed to variable responses [50,51].

Notably, other nutritional interventions, such as tart cherry juice, beetroot juice, curcumin, quercetin, and cocoa flavanols, have shown potential for reducing EIMD due to their antioxidant and anti-inflammatory properties [8,[52], [53], [54]]. However, variability in results across studies highlights the need to optimize supplementation strategies and better understand individual responses to these interventions. Similar inconsistencies observed with pomegranate supplementation emphasize the importance of standardized protocols and robust study designs.

Interestingly, 2 previous systematic reviews reported positive effects of pomegranate supplementation on EIMD markers; however, these were not meta-analyses [22,23]. In contrast, our systematic review and meta-analysis, which included 10 studies, provides a more comprehensive assessment but underscores the need for further research with larger sample sizes and standardized methodologies.

### Strengths and limitations

The primary strength of this study is that it is the first meta-analysis to evaluate the effects of pomegranate supplementation on EIMD markers. However, the heterogeneity among the included studies represents a notable limitation that affects the reliability of the findings. Variations in supplementation regimens (e.g., dose, duration), participant demographics (e.g., age, sex, training status), and exercise protocols (e.g., type, intensity) likely contributed to inconsistent results. Small study effects, such as the tendency of smaller studies to report larger treatment effects, could also bias the findings, even though publication bias was not detected through funnel plots and Egger’s tests. Additionally, methodological issues in some studies, such as lack of concealed allocation and ITT analysis, further limit the reliability of the conclusions. Because of the limited number of studies included, we were unable to perform subgroup analyses based on age, BMI, or sex. As a result, we cannot generalize the findings to specific subgroups like males, females, athletes, or nonathletes.

### Practical and future implications of research

Although this study did not find consistent effects of pomegranate supplementation on markers of EIMD, the significant decrease in CK and LDH amount immediately after exercise suggests potential benefits for recovery. Higher doses or longer durations of supplementation may be required to achieve more pronounced effects. Practitioners should exercise caution when recommending pomegranate supplementation for reducing muscle soreness or EIMD until further evidence is available. Future studies should focus on optimizing the dose, timing, and duration of pomegranate supplementation to better understand its potential role in mitigating EIMD and enhancing recovery. Suggested dose ranges could include 250–500 mL of pomegranate juice daily or its equivalent in concentrated extract form. Timing strategies should evaluate supplementation both pre- and postexercise, including administration 1–2 h prior to exercise, immediately after, or over extended periods (e.g., 1–2 wk). Additionally, further research should standardize exercise protocols to induce significant EIMD, such as high-intensity resistance training, eccentric exercises (e.g., downhill running or eccentric elbow flexion), or endurance activities like long-distance running or cycling. Standardizing these variables will allow for a clearer understanding of the conditions under which pomegranate supplementation may provide the greatest recovery benefits. Additionally, long-term studies investigating the effects of pomegranate supplementation on EIMD and recovery would provide valuable insights. Exploring combinations of pomegranate supplementation with other nutritional strategies or recovery interventions could further enhance its efficacy. By systematically addressing these factors, future studies can clarify the practical applications of pomegranate supplementation for athletes and active individuals.

In conclusion, the findings from this systematic review and meta-analysis suggest that pomegranate supplementation does not significantly influence most markers of EIMD, including muscle soreness, myoglobin, lactate, and CRP. However, a notable exception is a significant decrease in CK and LDH amounts immediately after exercise, which may indicate acute protective effects against EIMD. This protective effect is likely linked to the high polyphenol content in pomegranates, which are known for their potent antioxidant and anti-inflammatory properties.

## Author contributions

The authors’ responsibilities were as follows – SB, FK, MN: carried out the screenings and reviews; FK: carried out the analysis of the articles; SB, FK, MN, RB, MMH, SKR, DMC, FD: drafted and revised the manuscript; and all authors: read and approved the final manuscript.

## Data availability

The data that support the findings of this study are available from the corresponding author on request.

## Funding

The authors reported no funding received for this study.

## Conflict of interest

The authors report no conflicts of interest.
