# Supplementary material for: The Effects of Pomegranate Supplementation on Markers of Exercise-Induced Muscle Damage: A Systematic Review and Meta-Analysis
Source: Curr Dev Nutr. 2025 Jan 28;9(2):104560. doi: 10.1016/j.cdnut.2025.104560 (PMC11875170; doi:10.1016/j.cdnut.2025.104560)
Supplement: Multimedia component 1 [file mmc1.docx]

**The effects of** **pomegranate supplementation on markers of exercise-induced muscle damage: A systematic review and meta-analysis**

Saba Belyani

**Supplementary Table 1. pomegranate and Exercise Intervention Characteristics**

| **Source, Year** | **Intervention duration** | **Intervention characteristics** | **Placebo characteristics** | **Training Session** | **The time of measuring the variables** |
| --- | --- | --- | --- | --- | --- |
| Torregrosa-Garcia et al., 2019 (38) | 14 days | Composition per capsule: 375 mg of POMANOX® P30  with 30% punicalagins; total amount of punicalagins α + β per capsule: 112.5 mg  a total dose of 225 mg punicalagins | Maltodextrin | Cycling, with a training routine of 2-4 sessions/week, for at least 1-h/session. | CK: pre-exercise, post-exercise, 24h, 48h, 72h after exercise  CRP: pre-exercise, post-exercise, 24h, 48h, 72h after exercise  Lactate: pre-exercise, post-exercise |
| Trombold et al., 2009 (21) | 32 days | Pomegranate extract (POMx)  500 ml  Each 480-mL bottle  of POMx beverage contained 650 mg of pomegranate polyphenols consisting  of 95.5% ellagitannins, 3.5% ellagic acid, and 1% anthocyanins and 4 g of carbohydrate (maltodextrin and sucralose) | NM | 2 sets of 20 maximal eccentric elbow  flexion repetitions starting with the elbow at 50° of full flexion and ending at 170°. Repetitions were performed once every 15 s, with each repetition lasting 3 s. | Muscle soreness:  pre-exercise, post-exercise, 24h, 48h, 72h after exercise Myoglobin: pre-exercise, post-exercise, 24h, 48h, 72h after exercise  CK: pre-exercise, post-exercise, 24h, 48h, 72h after exercise  CRP: pre-exercise, post-exercise, 24h, 48h, 72h after exercise |
| Ammar et al., 2016 (35) | 15 days | Natraul Pomegranate Juice Supplementations  2000 ml  Each 500-mL of the tested POMj contained 2.56g of total polyphenol, 1.08g of orthodiphenols, 292.59mg of flavonoids and 46.75mg  of flavonols | NM | 2 sets of 3 reps at 85% weightlifting  and 3 sets of 2 reps at 90%. | CK: pre-exercise, post-exercise, 24h, 48h, 72h after exercise  CRP: pre-exercise, post-exercise, 24h, 48h, 72h after exercise  Muscle soreness:  pre-exercise, post-exercise, 24h, 48h, 72h after exercise  LDH: pre-exercise, post-exercise |
| Pranskuniene et al., 2020 (33) | 2 weeks | Pomegranate extract supplementation  1000 ml  20 mL dose (720 mg phenolic compounds measured as a Gallic acid equivalent according  to Folin-Ciocalteu) | NM | A 4-min warm up followed by a constant increase in the speed of 0.1 km/h every 6 s until fatigue. | Lactate: Before exercise, after exercise |
| May Crum et al., 2017 (20) | 11 days | Pomegranate extract (POMx) in capsule form  1000 mg | NM | A 5-min warm-up at 100 W, participants  completed four × 7-min stages of increasing workload (e.g., 150, 200, 250, 300 W) with expired air being collected in Douglas Bags during the last minute of each stage. | Lactate: before exercise, after exercise |
| L. Lamb et al., 2019 (34) | 9 days | Drink is Tart cherry juice (TC) and pomegranate juice (POM)  500 ml  Each serving of POM consisted of 250 mL of undiluted juice, whilst a serving of TC contained 30 mL of  concentrate diluted with 220 mL of water | Blackcurrant-flavored maltodextrin sports drink  500 ml | The eccentric exercise protocol consisted of 50 (five sets of 10) maximal voluntary eccentric contractions of the non-dominant elbow flexors on the isokinetic dynamometer. | CK: pre-exercise, post-exercise, 24h, 48h, 72h after exercise  Muscle soreness:  pre-exercise, post-exercise, 24h, 48h, 72h after exercise |
| Martinez-Sancheza et al., 2017(39) | 7 days | Mix of three different watermelon juice and a  concentrate of pomegranate from whole fruit enriched in L-citrulline and ellagitannins  200 ml | NM | A warm-up with 5-min of cycling on a cycle ergometer at 75 W followed by 10 repetitions at 50% of the perceived 1RM and active stretching exercises were performed.  In every session, the subjects lifted loads that allowed only 8 sets of 8 repetitions (8RM) to be performed with 2 min rest between sets of half squat. | Muscle soreness:  pre-exercise, post-exercise, 24h, 48h, 72h after exercise Myoglobin: pre-exercise, post-exercise, 24h, 48h, 72h after exercise  CK: pre-exercise, post-exercise, 24h, 48h, 72h after exercise  LDH: pre-exercise, post-exercise |
| Urbaniak et al., 2018 (36) | 2 months | Natural pomegranate juice (POM) supplementation  50 ml | composed of water, sugar, and grenadine, with  a colour and taste resembling that of the pomegranate  fruit juice | The athletes performed a controlled  2000-m rowing exercise test. | Myoglobin: pre-exercise, post-exercise, 24h, 48h, 72h after exercise  CK: pre-exercise, post-exercise, 24h, 48h, 72h after exercise |
| Trombold et al., 2011 (2) | 44 days | Pomegranate juice  250 ml twice daily | NM | The subjects performed 6 sets of 10 eccentric repetitions at 110% of their unilateral 1RM through a full range of motion. | Muscle soreness:  pre-exercise, post-exercise, 24h, 48h, 72h after exercise |
| Chedegani et al., 2014 (37) | 8 weeks | Natural pomegranate juice  250 ml | NM | Physical activity 3 times at week, 90 min running with 70% intensity VO_2max_ | CK: pre-exercise, post-exercise, 24h, 48h, 72h after exercise  LDH: pre-exercise, post-exercise  CRP: pre-exercise, post-exercise, 24h, 48h, 72h after exercise |

*Abbreviations: NM: Not mentioned, CK: Creatine kinase,* *CRP: C-reactive protein, LDH: Lactate dehydrogenase.*

**Supplementary Table 2. Quality assessment**

| Authors and Year of Publication | 1 | 2 | 3 | 4 | 5 | 6 | 7 | 8 | 9 | Total score |
| --- | --- | --- | --- | --- | --- | --- | --- | --- | --- | --- |
| Torregrosa-Garcia et al., 2019 | ✓ | 🗶 | 🗶 | ✓ | ✓ | ✓ | 🗶 | ✓ | ✓ | 6 |
| Trombold et al., 2009 | ✓ | 🗶 | 🗶 | ✓ | ✓ | ✓ | 🗶 | ✓ | ✓ | 6 |
| Ammar et al., 2016 | ✓ | 🗶 | 🗶 | ✓ | ✓ | 🗶 | 🗶 | ✓ | ✓ | 5 |
| Pranskuniene et al., 2020 | ✓ | ✓ | 🗶 | ✓ | ✓ | 🗶 | 🗶 | ✓ | ✓ | 6 |
| May Crum et al., 2017 | ✓ | 🗶 | 🗶 | ✓ | ✓ | ✓ | 🗶 | ✓ | ✓ | 6 |
| L. Lamb et al., 2019 | ✓ | ✓ | 🗶 | ✓ | ✓ | ✓ | 🗶 | ✓ | ✓ | 7 |
| Martinez-Sancheza et al., 2017 | ✓ | 🗶 | 🗶 | ✓ | ✓ | ✓ | 🗶 | ✓ | ✓ | 6 |
| Urbaniak et al., 2018 | 🗶 | 🗶 | 🗶 | ✓ | ✓ | ✓ | 🗶 | ✓ | ✓ | 5 |
| Trombold et al., 2011 | ✓ | 🗶 | 🗶 | ✓ | ✓ | ✓ | 🗶 | ✓ | ✓ | 6 |
| Bayat-Chadegani et al., 2015 | ✓ | 🗶 | 🗶 | ✓ | ✓ | ✓ | 🗶 | ✓ | ✓ | 6 |

*(1) Specified eligibility criteria, (2) Randomized participant allocation, (3) Concealed allocation, (4) Similarity of groups at baseline, (5) Blinding of all assessors, (6) Evaluated outcomes in 85% of participants, (7) Intention-to-treat (ITT) analysis, (8) Reporting of statistical comparisons between groups, (9) and Point measures and statistics of variability.*
